# Microbial community diversity and geochemistry inform bioremediation of molybdenum-contaminated groundwater

**DOI:** 10.1128/aem.00988-25

**Published:** 2025-12-05

**Authors:** Natalia Malina, Rodney Tollerson, Shifat J. Monami, Elyssa Rivera, Ming-Kuo Lee, Laura D. Bilenker, Ann Sullivan Ojeda

**Affiliations:** 1 Department of Geosciences, Auburn University 1383, Auburn, Alabama, USA; 2 Department of Chemistry and Biochemistry, Florida Atlantic University 1782, Boca Raton, Florida, USA; 3 Department of Biological Sciences, Auburn University 1383, Auburn, Alabama, USA; Washington University in St. Louis, St. Louis, Missouri, USA

**Keywords:** groundwater, molybdenum, bioremediation, metagenomics, sequestration

## Abstract

*In situ* remediation of groundwater at coal combustion product (CCP) sites can be challenging for elements such as molybdenum (Mo), which do not respond well to commonly used treatment. This research was initiated to improve the understanding of geochemistry and microbial diversity associated with a Mo plume at a CCP site toward the development of an *in situ* treatment scheme. Diffusive microbial samplers were designed and deployed at the study site for 9 weeks. Afterward, geochemical and community analyses were used as the basis to understand how microbial communities respond to elevated Mo concentrations within a plume. Our results show that the Mo and other constituents within the plume do not reduce the diversity of the community, in contrast to trends observed at other industrial sites with metals and metalloids in groundwater. Interestingly, bacteria of the order *Burkholderiales* were higher in abundance in wells where Mo >0.3 mg/L, and several sulfate-reducing bacteria were less abundant but not absent. Molybdenum sequestration experiments were also performed with sulfate-reducing bacteria enriched from groundwater samples collected at the site. The results show that *Desulfomicrobium escambiense* played a major role in Mo sequestration and activated a detoxification mechanism. This process involved the sequential activation of periplasmic heavy metal sensors, followed by the activation of *atpE* ATP synthase, which may function as an exporter of Mo to form Mo-S species in the periplasm of the cell. The results provide important considerations for bioremediation potential in groundwater settings impacted by Mo, especially those who seek to stimulate sulfate-reducing bacteria for Mo sequestration in biogenic sulfide solids.

**IMPORTANCE:**

Bioremediation of contaminated sites has become popular for chlorinated hydrocarbons, but it has not been widely applied to inorganic constituents outside of arsenic. Here, we show the potential for the development of geochemistry-informed bioremediation technologies of Mo-contaminated groundwater by leveraging Mo-tolerant communities despite the suppression of sulfate reduction by Mo.

## INTRODUCTION

Coal has been a globally dominant source of energy over the past century. Coal combustion product (CCP) includes bottom ash, fly ash, boiler slag, and flue gas desulfurization products that are either beneficially reused or stored in landfills or surface impoundments (1). Where there has been a release of leachate from a CCP landfill or impoundment, groundwater can contain elevated concentrations of inorganic elements, some of which are difficult to treat *in situ*, including molybdenum (Mo). Bioremediation is investigated here as an *in situ* remediation strategy, whereby microorganisms (either naturally occurring or introduced to the site) are stimulated to sequester or transform constituents of interest to achieve site clean-up goals.

Bioremediation potential is dependent on biogeochemistry within the site. Groundwater microbial ecology is unique because of the exclusion of light, low oxygen conditions, and relatively poor nutrient availability. Groundwater systems are typically organic carbon-limited unless they are tightly coupled to the land surface so that organic matter originating from the surface is efficiently transported to the groundwater (2). Inorganic carbon is typically available in excess in groundwater so that a sufficient flux of electron donors (H_2_, NH_4_^+^, NO_2_^-^, HS^-^, S^0^, S_2_O_3_^2-^, CO, or Fe^2+^) and electron acceptors (O_2_, NO_3_^−^, SO_4_^2−^, S^0^, S_2_O_3_^2−^, Fe^3+^, and CO_2_) can sustain chemoautotrophic and chemolithotrophic communities. Exposure to constituents leached from CCPs, including certain metals and metalloids, can affect groundwater community assemblages. Elements like As and Cr can damage cell membranes, disrupt cellular function, and cause DNA damage (3). At the community level, this can be reflected in a loss of microbial and metabolic diversity, favoring the growth of resistant species (4, 5). Native enrichment in microorganisms (and absence of others) in the presence of CCP plumes can provide valuable insights into viable bioremediation strategies, as it points to tolerant (or intolerant) taxa, which can further be probed to understand biological response and function during exposure. Ultimately, leveraging biological function is the basis of effective bioremediation.

In environmental systems, Mo influences microbial community structures. Mo is a cofactor for many enzymes and, therefore, an essential micronutrient for biological growth (6). Since Mo is limited in its bioavailability in natural systems, excess Mo uptake can limit biological function (7). Some studies investigate Mo because it co-occurs with other metals like As, Cd, and U in mine-waste-affected soil and water (8, 9), but in these cases, multiple stressors make the effect of elevated Mo concentrations difficult to isolate. In high-Mo environments, biological growth coupled to Mo-transformation falls into one of two classes: reduction to molybdenum blue, a polymer of phosphate and Mo (10, 11), or Mo-reduction accompanied by sulfide production to form insoluble MoS_2_(12).

Mo is typically found as molybdate (MoO_4_^2-^) in environmental settings, and sulfate-reducing bacteria (SRB) utilize sulfates as a terminal electron acceptor, forming hydrogen sulfides that can subsequently react with Fe or Mo to form a biogenic pyrite (12) or molybdenite (13). Molybdate is a structural analog of sulfate and competes to enter cells via sulfate transporters at high concentrations and inhibits the production of sulfides (14, 15). Inhibition of SRB respiration with Mo is applied to prevent microbial sulfidogenesis in several industrial processes (16). However, some SRB tolerate elevated concentrations of molybdenum and are used for molybdenum sequestration (7). The concentration of molybdate that inhibits microbial function varies widely by species. Sulfate reduction in Mo-sensitive species can be suppressed at concentrations as low as 3 µM (0.3 mg/L Mo) (17, 18), and sublethal doses (< 2 mM molybdate) can lead to sequestration of Mo in sulfidic solids (19–21). Conversely, species that produce Mo-blue have been documented to grow with molybdate concentrations as high as 100 mM (22).

The mechanisms of biotic Mo sequestration involve complex interactions between microbial communities and minerals. There are several possible mechanisms of biotic Mo sequestration: (i) precipitation due to biogenic mineralization (21) or adsorption on the biogenic pyrite formed by SRB in the presence of iron (Fe) (23), (ii) microbial cell uptake by binding to intracellular ligands and sedimentation upon microorganism death (23), and (iii) binding of biogenically formed molybdenum sulfide to the periplasmic proteins and amino acids (7, 23). There are many gaps in understanding how these mechanisms co-occur, affect SRB functions, or compete for biogenically formed sulfides in the presence of Fe and Mo. Biosequestration of the metals and metalloids by SRB is a promising treatment technique for contaminated groundwater, acid mine tailing wastes, and wastewater (24, 25). Therefore, a fundamental understanding of metal biosequestration is necessary for the development of bioremediation techniques.

In this study, we focus on the geochemistry and microbial diversity of a carbonate aquifer impacted by a release of CCP leachate to groundwater and characterize Mo sequestration by SRB isolated from groundwater. Mo is an essential element for enzymatic function but is limited in most environmental systems; however, in this study, the groundwater is enriched in Mo, creating a unique habitat to control microbial dynamics. Our goal was to apply a holistic approach to understand how the release of CCP leachate containing Mo affects subsurface geochemistry and microbial dynamics and to identify the prevailing mechanism of Mo sequestration by isolating SRB from contaminated groundwater SRB. Our research questions were as follows. (i) How do groundwater microbial communities respond to groundwater with different levels of Mo? (ii) What are the dominant biogeochemical processes present? (iii) What is the predominant mechanism of Mo sequestration by SRB in the presence of Fe? These insights can elucidate the bioremediation potential for Mo in groundwater at CCP sites.

## MATERIALS AND METHODS

### Study area description

The study area is a CCP impoundment site, referred to as EPRI 01157, which has been in operation since the 1960s (general site overview in Fig. S1). The impoundment is unlined and is bounded on the downgradient side. The site is underlain by two hydrogeologic units: a clay-rich overburden on top of a dolomite and limestone aquifer that is part of the larger Valley and Ridge Aquifer System. Groundwater flow rates range between 0.03 and 0.16 ft/d (0.009 and 0.048 m/d). Groundwater quality at the site is monitored, but for this effort, we have focused attention on the seven monitoring wells (MW) shown in Fig. S1. Groundwater elevations fluctuate seasonally, but groundwater flows toward the river. The MWs selected for this study represented a range of Mo concentrations (below detection to 2.883 mg/L), and two MWs served as controls: one (MW-6) upgradient of the Mo plume and a second (MW-7) across the river, where groundwater has not been affected by the release from the impoundment.

### Groundwater collection and chemistry

At each well, groundwater was purged using a bladder pump connected to a flow-through multi-parameter EXO2 water quality sonde that measured temperature (°C), dissolved oxygen (DO, mg/L), pH, oxidation-reduction potential (ORP, mV), and conductivity (μS/cm). Wells were purged until field parameters stabilized. After stabilization, water quality parameters were recorded, and groundwater samples were collected in 1 L bottles after rinsing with groundwater three times prior to collection. Water samples were filtered using a 0.45 µm syringe filter and then split into two aliquots, with one acidified with trace-grade HNO_3_ (to 3% or 30 g/L nitric acid) for preservation following standard procedures (26) and one sent for anion analysis without acidification. All water samples were collected in acid-cleaned high-density polyethylene bottles with zero headspace. They were stored in ice-packed coolers (with a temperature around 5°C) immediately after collection. In the lab, samples were stored at 4°C prior to analysis.

Major cation and trace element concentrations of groundwater were measured using an Agilent 7900 quadrupole inductively coupled plasma mass spectrometer (ICP-MS) at Auburn University following a modified version of EPA Method 200.8. Anion (chloride, sulfate, nitrate, and phosphate) concentrations were measured using a Dionex DX500 ion chromatograph (IC) at the University of Georgia.

Geochemist’s Workbench (27), a geochemical modeling software, was used to calculate the speciation of Mo and determine the dominant Mo species under various Eh-pH conditions at the site. Geochemist’s Workbench was also used to construct a Piper diagram to determine hydrochemical facies.

### Subsurface microbial sampling

We constructed a diffusive microbial sampler (DMS) to passively allow microbial communities to colonize a substrate and to maintain the downhole aquifer conditions prior to microbial analysis. The DMS was inspired by a United States Geological Survey prototype that was used to sample a coal-bed methane consortium (28), as well as previous efforts by our team to collect biogenic minerals formed in groundwater wells (29). A detailed description of the DMS construction is included in the supplemental material, Section 2, and Fig. S3. Briefly, a Snap Sampler (QED Environmental) was modified to contain a sand packet in the sample vials on which native microbial communities could colonize. At the end of the deployment, samples were retrieved by pneumatic closure of the vials, thus maintaining groundwater conditions within the sample.

The DMSs were deployed in seven monitoring wells near the CCP impoundment with 2 Snap Sampler units per well for duplicate measurements, referred to hereafter by designations of T or B for top or bottom sample, respectively. DMSs were positioned in the center of the screened interval of each well. Two wells were chosen as controls: MW-6 is outside of the plume flow path but is near the other MWs, and MW-7 also serves as a control with low Mo, but it is about 1 km away from the well grouping near the plume and across the river. The DMSs were deployed on 10–13 April 2023 and retrieved on 12 June 2023, for a total deployment time of 9 weeks. Of the 14 samples, 11 sample vials successfully closed downhole with the pneumatic closure, and three were not closed upon retrieval (2T, 3T, and 6T). At each well, at least one sample maintained under downhole conditions, ensuring that groundwater conditions were preserved at each site. Sample vials that did not close downhole were closed immediately upon retrieval; it should be noted that there was no aqueous phase associated with samples that did not close downhole because the groundwater drained from the vial as the sampler was retrieved. These samples were exposed to oxygen for approximately 10 min prior to closure in an oxygenated environment at the well head. Samples were transported to the laboratory in secondary bags containing several oxygen scavenging packets and on ice (4°C). At the lab, samples were stored in a Type C Coy anaerobic chamber that maintains 0–1 ppm O_2_ and immediately prepared for microbial analysis.

### Microbial community analysis

For each sample, sand (~8 g) and water (~40 mL) were transferred from the sampling vial to 60 mL sterile wide-mouth HDPE bottles. Samples were split for microbial analysis, so approximately ~4 g of sand and ~20 mL water (when present) were transferred to sterile HDPE bottles and sent to Molecular Research Laboratory (Shallowater, TX) for analysis. A blank sample using sterile 18 MΩ water and sand was prepared in parallel with the samples and sent for analysis.

The 16S rRNA gene V4 variable region PCR primers 515/806 were used in 30–35 cycles polymerase chain reaction (PCR) using the HotStarTaq Plus Master Mix Kit (Qiagen, USA) under the following conditions: 95°C for 5 min, followed by 95°C for 30 s, 53°C for 40 s, and 72°C for 1 min, after which a final elongation step was performed at 72°C for 10 min. After amplification, PCR products were checked in 2% agarose gel to determine the success of amplification and the relative intensity of bands. Samples were multiplexed using unique dual indices and were pooled together (e.g., 100 samples) in equal proportions based on their molecular weight and DNA concentrations. Pooled samples were purified using calibrated Ampure XP beads. Then, the pooled and purified PCR products were used to prepare an Illumina DNA library. Sequencing was performed at Molecular Research Laboratory (Shallowater, TX, USA) on a MiSeq following the manufacturer’s guidelines. Sequence data were processed using the analysis pipeline of Molecular Research Laboratory (Shallowater, TX, USA). In summary, sequences were joined, sequences < 150 base pairs (bp) removed, and sequences with ambiguous base calls removed. Sequences were quality filtered using a maximum expected error threshold of 1.0 and dereplicated. The dereplicated or unique sequences were denoised; unique sequences that were identified with sequencing and/or PCR point errors were removed, followed by chimera removal, thereby providing a denoised sequence or zero-radius operational taxonomic unit (zOTU). The final zOTUs were taxonomically classified using BLASTn against a curated database derived from NCBI (www.ncbi.nlm.nih.gov) and compiled into taxonomic levels into both “counts” and “percentage” files.

### Sequence data processing

Microbial community data analyses were performed in Microbiomeanalyst v 2.0 (30, 31). Overall, 1,060 zOTUs were identified in the samples. Taxa dominant in the blank sample were removed from the analysis. Data were filtered using a minimum count threshold of 4 and a 20% prevalence in samples; a total of 463 low-abundance features were removed. Data were not rarefied and were scaled for analysis using total sum scaling.

### Isolation and enrichment of the culture

Upon delivery to the laboratory, the SNAP samplers were opened in an anaerobic chamber, and half of the sand was transferred to a 125 mL serum bottle with ATCC 1249 Modified Baar’s medium. The samples were incubated for 2 weeks under anaerobic conditions at room temperature. At the end of 2 weeks, the samples were visually turbid, and 100 μL of the solution was transferred to a 15 mL serum bottle containing 10 mL of Baar’s medium. The process was repeated five times to enrich SRB. On the fifth enrichment, ~5 mL of the enriched media from each bottle was transferred to a sterile wide-mouth HDPE bottle and sent to Molecular Research Laboratory (Shallowater, TX) to analyze the microbial community in the samples following the procedure described in the Microbial Community Analysis section above.

### Molybdenum sequestration experiments

The Mo sequestration experiment was performed using the enriched microbial community. The microbial cells were washed with a non-sulfate-containing Baar’s medium prior to the sequestration experiment. The composition of the non-sulfate-containing Baar’s medium was similar to that used for the enrichment of SRB, with no addition of MgSO_4_ and CaSO_4_. Enriched cultures were transferred to a 15 mL centrifuge tube in the anaerobic chamber and centrifuged at 4,000 rpm for 15 min. The supernatant was carefully removed with the pipette, 10 mL of the fresh non-sulfate containing Baar’s medium was added to the centrifuge tube and mixed manually. The washing procedure was repeated twice. The culture was used in the experiments within 24 h after washing.

The Mo sequestration experiment was performed in 125 mL serum bottles in Baar’s medium. Mohr’s salt (ammonium iron(II) sulfate) was used as a reducing agent, and the final concentration of Fe in the medium was 20 ppm; 500 mL of resazurin (0.1% wt/vol) was added to the 1 L of medium to indicate that anaerobic conditions were maintained during the duration of the experiment. Sodium molybdate was used as the source of Mo. Stock solution of sodium molybdate (3,000 ppm as Mo) was added to 100 mL Baar’s medium to reach a final concentration of 3.0 ppm Mo. The experiment was performed in triplicate. A set of control experiments was prepared as (i) a biotic control where no Mo was added, (ii) an abiotic control where the enriched culture was not added, and (iii) killed controls where 5 mL of sodium azide (1% wt/vol) was added to inhibit microbial growth. Control experiments were performed in parallel with treatment groups. All experiments were performed at room temperature and lasted ~168 h.

Aliquots from the experiments and controls (~5 mL) were collected every 24 h using a sterile syringe through the septa, and the samples were filtered through a 0.22 mm syringe filter to remove microbial culture from the solution. The filtrate was divided into two parts. One aliquot was preserved with zinc acetate solution, and the other with nitric acid for sulfide and metal analysis. Sulfide was measured using the reagent colorimetric method by the Cary-UV VIS system (Agilent, USA) (32). Iron and Mo concentrations were measured using an Agilent 7900 quadrupole inductively coupled plasma mass spectrometer (ICP-MS) at Auburn University, following a modified version of EPA Method 200.8. The precipitates that formed in the experiment were analyzed using a Zeiss EVO-10 scanning electron microscope (SEM) equipped with an energy-dispersive X-ray spectroscopy (EDS) detector at the Engineering Analytical Laboratory of Auburn University, following the procedure described in supplemental material, Section 3.

### Preparation of RNA-seq libraries

Following the procedure described for the sequestration experiment, six 125 mL serum bottles were prepared and filled with Baar’s medium. Three of the bottles were dosed with Mo to reach the final concentration of 3 ppm; three microcosms proceeded without dosing of the Mo. Gene expression was measured in triplicate at 60 h after Mo treatment. Samples were collected by anaerobic centrifugation at 10,000 × *g* for 10 min at room temperature. Cell pellets were resuspended in 300 µL TRI Reagent (Zymo Research), and RNA was purified using the Direct-Zol RNA Microprep kit (Zymo Research) according to the manufacturer’s instructions. After purification, ribosomal RNAs and other abundant non-coding RNAs were depleted using the pan-Bacteria riboPOOLS rRNA depletion kit (siTOOLS) and concentrated using an Oligo Clean and Concentrator kit (Zymo Research). The resulting rRNA-depleted RNA was quantified using a Qubit fluorimeter (Invitrogen), and the libraries were prepared using NEBNext Ultra II Directional RNA Library Prep Kit for Illumina (New England Biolabs). Libraries were barcoded, pooled, and sequenced with an Illumina MiniSeq with 300-cycle High Output reagents at Auburn University.

### RNA-seq analysis

Raw RNA-seq fastq files were analyzed for quality using FastQC (Babraham Bioinformatics), and those that were below a quality score of 20 were trimmed with BBDuk (Joint Genome Institute). Trimmed reads were mapped using BBMap (JGI) and counted using featureCounts (33). The counts were then analyzed using DESeq2 (34). Plots were made using EnhancedVolcano.

### Transmission electron microscopy (TEM)

TEM analysis was performed by the University of Georgia using a JEOL JEM2100 PLUS (JEOL USA, Inc., Peabody, MA). Control and experimental cultures were fixed in 2.5% glutaraldehyde in 0.1M Cacodylate-HCl buffer, pH 7.2. The samples were washed several times in 0.1M Cacodylate-HCl buffer, pH 7.2 with light centrifugation before agar-enrobing each sample in 4% Noble Agar, aqueous. Once the agar-bacteria pellets were set, they were placed back in the buffer before post-fixation in 1% osmium tetroxide in 0.1M Cacodylate-HCl buffer, at pH 7.2 for 1 h. The pellets were washed several times in deionized water, dehydrated in an ethanol series (50%, 75%, 95%, 95%, and 100%), then cleared in two changes of propylene oxide. The sample pellets were infiltrated with 2:1, 1:1, and 1:2 mixtures of propylene oxide and Mollenhauer’s Epon-Araldite plastic mixture: 1 h for the 2:1 mixture, 2 h for the 1:1 mixture, and overnight in the 1:2 mixture. The embedded samples were polymerized in a 70°C–80°C oven overnight.

During ultramicrotomy, 60–70 nm sections were obtained and placed on lacey carbon grids and on 200-mesh copper grids. The sections placed on the lacy grids were sans post-stained and imaged with a Hitachi SU9000 low kV STEM operating at an accelerating voltage of 30 kV. In this imaging scheme, bright-field images and EDS data, collected with an Oxford Ultim Extreme Detector, were obtained. Sections on the 200-mesh copper grids were post-stained with 2% aqueous uranyl acetate and Reynolds lead citrate prior to viewing with the JEOL JEM-2100PLUS TEM operating at an accelerating voltage of 120 kV. Images were acquired using an AMT NanoSprint15L-MarkII High Sensitivity sCMOS TEM Camera with a resolution of 5,056 × 2,960 pixels.

## RESULTS AND DISCUSSION

### Geochemical setting

The hydrochemical facies for background groundwater at the site was Ca-Mg-HCO_3_, which is largely reflective of the dolomitic aquifers (Fig. S2). The control MW-7 contains more siliciclastic material, reflected in the lower pH value, but it is still within the normal range of a carbonate aquifer and shares similar hydrochemical facies as the control sample from MW-6. MW-1 was within the plume and had the highest Mo concentration, followed by MW-2, MW-3, and the hydrochemical facies shifted to Ca-Mg-SO_4_ dominant with higher total dissolved solids concentrations. MW-4 and MW-5 showed lower concentrations of Mo, and the hydrochemical facies showed higher proportions of HCO_3_. Sulfate and total dissolved solids (TDS) are also elevated in MW-1, MW-2, and MW-3, reflecting the CCP source for the plume (Table 1). Carbonate aquifers typically have high dissolved oxygen (DO) content (35), creating aerobic microbial environments. However, suboxic and anoxic conditions (DO <0.5 mg/L) were present in wells MW-1 and MW-3, whereas MW-5 and MW-2 were approaching suboxia (0.6–0.7 mg/L). Interestingly, MW-4 was aerobic (DO = 1.21 mg/L) with the highest total organic content (TOC) (5.68 mg-C/L TOC), whereas other wells within the plume contain low TOC (BDL-1.80 mg-C/L).

**TABLE 1:** Summary of groundwater chemistry parameters^*e*^

Well	pH^*a*^	Eh^*b*^ (v)	Conductivity^*a*^ (μS/cm)	DO^*a*^ (mg/L)	Sulfide^*c*^ (mg/L)	Sulfate^*d*^ (mg/L)	Nitrate^*d*^ (mg/L)	Chloride^*d*^ (mg/L)	TOC^*c*^ (mg-C/L)	Mo^*d*^ (mg/L)
MW-1	9.24	0.0740	2,187.81	0.32	0	498.40	BDL	468.13	1.17	2.883
MW-2	7.80	0.0799	1,098.43	0.74	0	634.92	BDL	19.52	BDL	0.812
MW-3	7.97	0.1205	945.91	0.20	1.00	458.45	BDL	16.38	1.80	0.322
MW-4	8.13	0.0074	927.84	1.21	3.00	129.90	BDL	103.76	5.68	0.039
MW-5	8.19	0.0242	438.34	0.58	1.00	39.59	BDL	7.34	1.29	0.010
MW-6 (control)	7.71	0.0316	398.21	1.02	0	28.06	BDL	13.83	BDL	0.014
MW-7 (control)	6.38	0.4096	101.36	7.68	0	1.93	0.88	3.84	BDL	BDL

^*a*^ *In situ *measurement during DMS deployment.

^*b*^ Oxidation-reduction potential was measured in the field and converted to Eh following the probe manufacturer’s instructions.

^*c*^ Values reported by the site managers in 2022.

^*d*^ Measured from samples collected during DMS deployment.

^*e*^ BDL = below detection limit: nitrate = 0.15 mg/L; TOC = 1 mg/L; Mo = 0.0001 mg/L.

**Fig 1:**
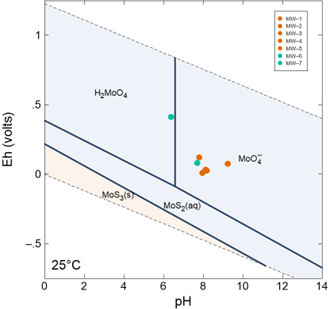
Pourbaix diagram of the Mo-S system using the geochemical data presented in Table 1. For modeling purposes, Mo concentration was set to 0.6 ppm at 25°C. Green symbols indicate control wells, and orange symbols indicate plume wells.

The geochemical fate and transport of Mo and other trace elements are governed by redox conditions and pH (27). In oxic environments, Mo is typically found as molybdate (MoO_4_^2-^), which is soluble in water, and its sorption to sediment is dependent on the mineral matrix and pH, with the highest adsorption of MoO_4_^2-^ observed at low pH values (3–5) onto oxides and clay minerals with positive surface charges (36, 37). Mo also forms complexes with organic matter, sequestering Mo in organic-rich sediments (38). Similar to oxides and clays, complexation with organic matter is pH-dependent, with the highest adsorption at pH 4–5 (38, 39). Geochemical modeling predicted that molybdate predominated under the field Eh-pH conditions in all wells (Fig. 1). Insoluble MoS_2_ solids would only exist under reducing conditions. The low organic matter content of the aquifer (Table 1), dominant oxic-suboxic conditions, and high pH buffering capacity of the dolomite-limestone aquifer confirm the mobility of Mo in the form of MoO_4_^2-^ in the subsurface.

### Microbial community characteristics

**Fig 2:**
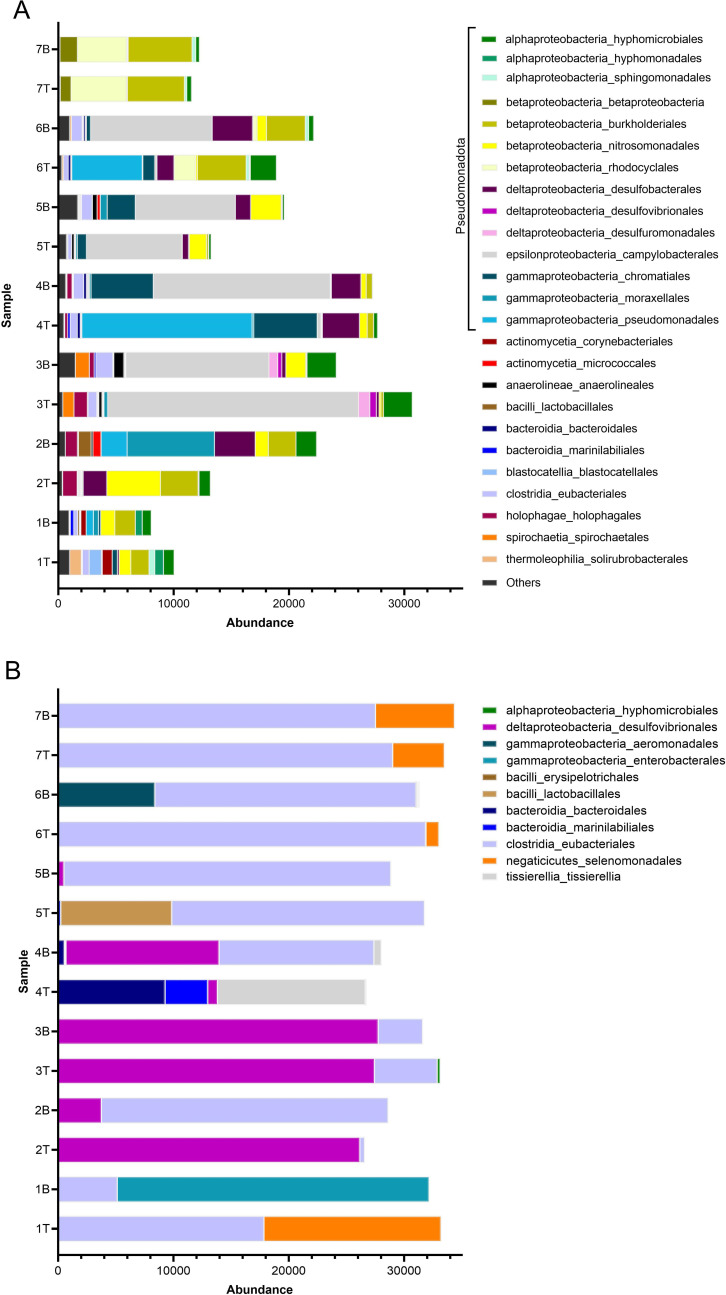
Bacterial community abundance (>1%) at the order level prepended by the class: (**A**) groundwater isolates; (**B**) after enrichment for sulfate-reducing bacteria in Baar’s media. Sample labels correspond to the site description in Fig. S1.

Bacteria accounted for over 99% of the zOTUs (zero-radius OTUs) in all samples except MW-1B, MW-7T, and MW-7B, where T and B refer to the top and bottom sample vials in the DMS assembly, respectively. In these samples, Archaea accounted for ~1%–2% abundance. The low abundance of Archaea could be due to the primers selected for amplification, which are known to favor bacteria (40). The community composition classified by order with names prepended by taxonomic class for each sample is shown in Fig. 2A. *Pseudomonadota (formerly Proteobacteria*) are the most abundant with high abundances of *Alpha-*, *Beta-*, *Delta-*, *Epsilon-*, and *Gammaproteobacteria* accounting for between 53% (MW-1T) and 99% (MW-7B) of the bacteria present. *Deltaproterobacteria* of the classes *Desulfobacterales, Desulfovibrionales, and Desulfuromonadales* (purple colors in Fig. 2A) were identified as SRB in the microbial community present in the groundwater.

The diversity in most samples was high, ranging from 0.75 to 0.92 in the Simpson index, where the maximum value is 1 (Fig. S4). Shannon entropy generally ranged from 2 to 3; similar Shannon entropy values represent similar ranges of richness and evenness across sample sites. Most duplicate samples (T and B, respectively, of the same well) showed similar diversity (Fig. S4); however, sample 3T (not closed downhole) showed a noticeably lower diversity than sample 3B, which was successfully closed downhole. Conversely, the other open/closed sample pairs (2T/B and 6T/B) showed similar metrics; hence, the wide range in alpha-diversity metrics observed for MW-3 cannot be solely explained by differences in sample preservation. Additionally, it is noted that the methodological bias in the choice of primers could underrepresent the abundance and diversity of Archaea in the native community, which would in turn affect the diversity metrics here.

We also determined the beta diversity, highlighting the potential for dissimilarity between samples using principal coordinate analysis (PCoA) of the Bray-Curtis distance calculated between sample pairs (Fig. S5). Variance in the first two PCoA dimensions accounted for 48.2% of the total variance in the data set. Control samples from MW-7 (across the river from the impoundment) were separated from samples collected at the CCP site. Ordination revealed no significant differences (PERMANOVA, *P* > 0.05) between samples at the CCP site, regardless of sample closure downhole. The degree of hydraulic connectivity between sites (degree of connected fractures in this case) is likely to affect the dispersion of microbes and drive differences in community assemblages. The low-permeability matrix in carbonate-rock aquifers is likely to have limited microbial biomass transport, similar to other materials; therefore, microbial biomass mostly accumulates in fractures. In that case, differential transport among bacterial strains could contribute to field-scale biodiversity (41). Therefore, a connected system is likely to reduce spatial heterogeneity of microbial communities (4, 42), which also contributes to the difference observed between the impoundment site and the control site across the river.

Relationships between community members were also investigated using the Ward clustering method (Fig. S5). Overall, duplicate samples (T and B) tended to cluster together, indicating similarities between microbial communities. Interestingly, plume wells with higher conductivity and sulfate concentrations (MW-1, MW-2, MW-3, and MW-4) showed a first-level separation. Another point to notice is that there is no clear cluster of samples that did not close downhole (2T, 3T, and 6T), but these samples showed a statistically lower abundance of *Bacillota (formerly Firmicutes*) compared with plume wells that closed downhole (Wilcoxon test, *P* = 0.009). This provides some evidence that exposure to oxygen did impact the microbial community, but it is also possible that the overall high microbial diversity observed at the site accounts for the difference observed.

For the wells within the plume, the low abundance of DO, nitrate, and Fe (III) suggests that sulfate would act as an electron acceptor. Elevated sulfate concentrations in the wells within the plume range from 39 to 630 mg/L, but low H_2_S concentrations (0–3 mg/L) suggest that the sulfur cycle could be limited by sulfate/sulfur reduction or consumed by other elements to form sulfide minerals.

### Impact of Mo on the microbial community

Examining the trends in microbial communities in the groundwater plume can provide insight into how Mo potentially affects community assemblage and the potential for Mo-adaptation within the community. In anoxic conditions, molybdate is a structural analog of sulfate and competes with sulfate for binding in sulfate-reduction pathways and inhibits sulfate reduction as a consequence (43, 44). The high Mo concentration coincides with the accumulation of sulfate in the aquifer system of the studied area, suggesting the inhibition of sulfate reduction. It should be noted that oxic and highly alkaline field conditions may also suppress bacterial sulfate reduction. We further investigate the effect of Mo on community structure through the lens of community diversity indices, enrichment in taxa, and gene expression as a function of Mo.

**Fig 3:**
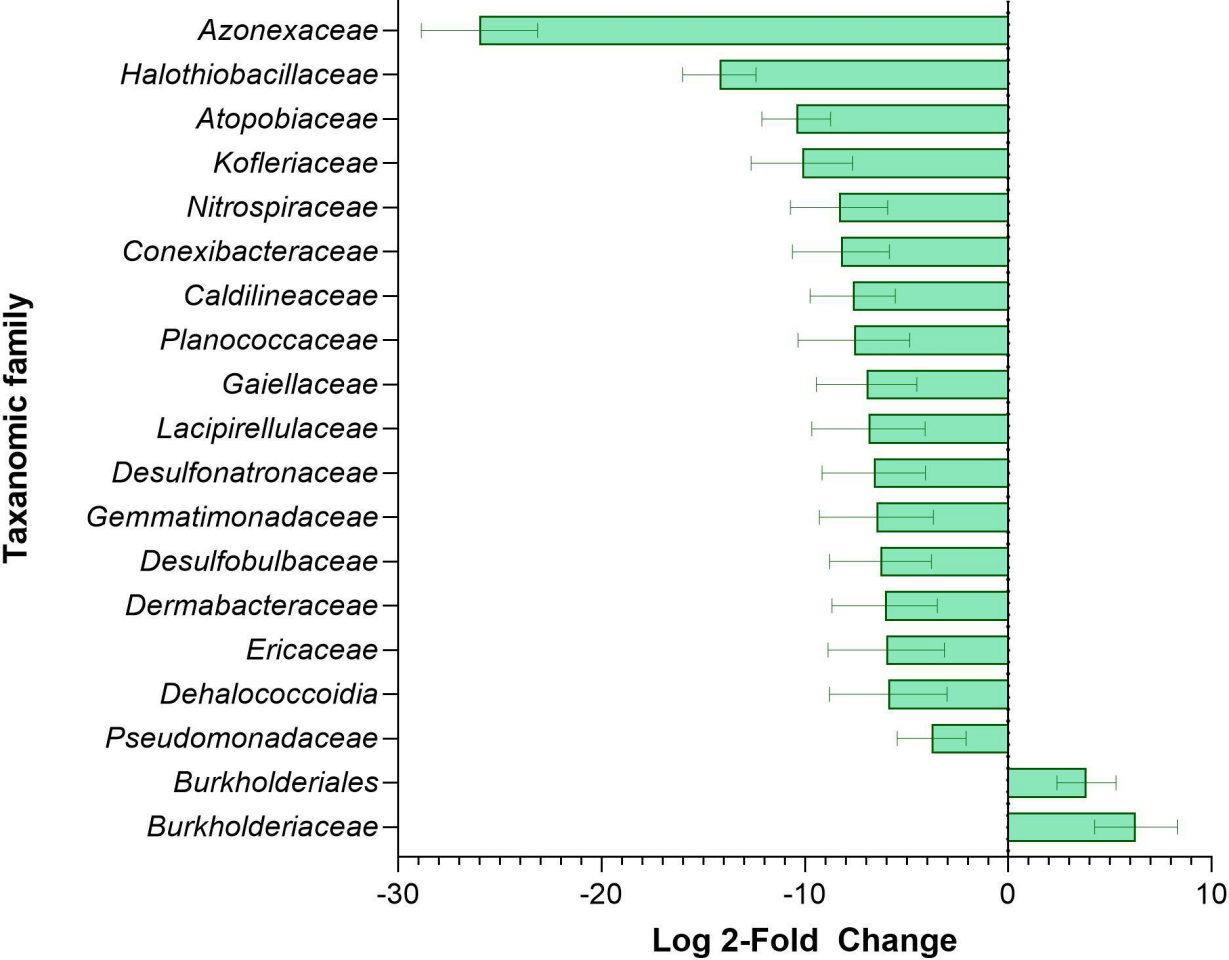
The log2 FC in taxonomic family in high (>0.3 mg/L) versus low (<0.3 mg/L) Mo wells.

We observe that the relative abundance of microbes identified in the high-Mo wells was similar to wells with low-Mo and the control wells (MW-6 and MW-7). Furthermore, the high-Mo wells did not display lower alpha-diversity compared with other samples within the plume, nor did these wells display a distinct community profile in beta-diversity or the cluster analysis. To analyze the differences between taxa, we used DESeq2 to compare taxonomic families between high- (MW-1, MW-2, and MW-3) and low-Mo wells (MW-4, MW-5, and MW-6; Fig. 3). MW-7 was omitted from this grouping because of its distinction in beta-diversity described above. *Burkholderiales* were present in higher abundance in high-Mo wells, showing a log2FC of +6.23. *Burkholderiales* were previously shown to be in higher abundance in sediment from non-CCP sites contaminated with U, Tc, chlorinated solvents, and other metals (5, 45), Cd (46), As, Cd, Cr, Cu, Ni, Pb, and Zn (47). *Burkholderiales* also possess a wide range of metal-resistant genes that manage the response to stressors (48, 49), but there is no evidence yet that Mo induces similar stress responses as other metals or metalloids (50, 51). A range of taxa was found to be statistically lower in abundance in high-Mo wells, including sulfate-reducing bacteria of the family *Desulfobulbaceae* and *Desulfonatronaceae*, which again is consistent with suppression of sulfate-reduction metabolic pathways by molybdate. Overall, the release from the CCP unit had some impact on the microbial community, but not to the extent observed for other metals and metalloids at other types of sites, as seen in recent examples (8, 52, 53) and described in the more general review contained in Griebler and Lueders (4).

### Groundwater microbial community enrichment

The enrichment of groundwater samples under sulfate-reducing conditions limited the microbial community to 10 unique orders (Fig. 2B). There is a distinguished difference between the microbial communities depending on the Mo concentration in the groundwater. Low-Mo wells showed enrichment with *Eubacteriales*. In contrast, *Desulflovibrionales* are predominant in high-Mo wells MW-2 and MW-3. The microbial community of MW-1 that has the highest Mo concentration is enriched by *Enterobacteria*, *Eubacteriales*, and *Selenomadales*. We chose the enrichment culture from MW-3 for use in the Mo sequestration experiment, based on the high abundance of this microbial community in most high-Mo wells, which indicates a potential tolerance of this microbial strain to the Mo concentrations at the site. More generally, *Desulflovibrionales* were used for the biological treatment of the wastewater due to their tolerance to the high concentrations of Cr, Cu, Mn, Ni, and Zn and their ability to precipitate these metals from solution (54).

**Fig 4:**
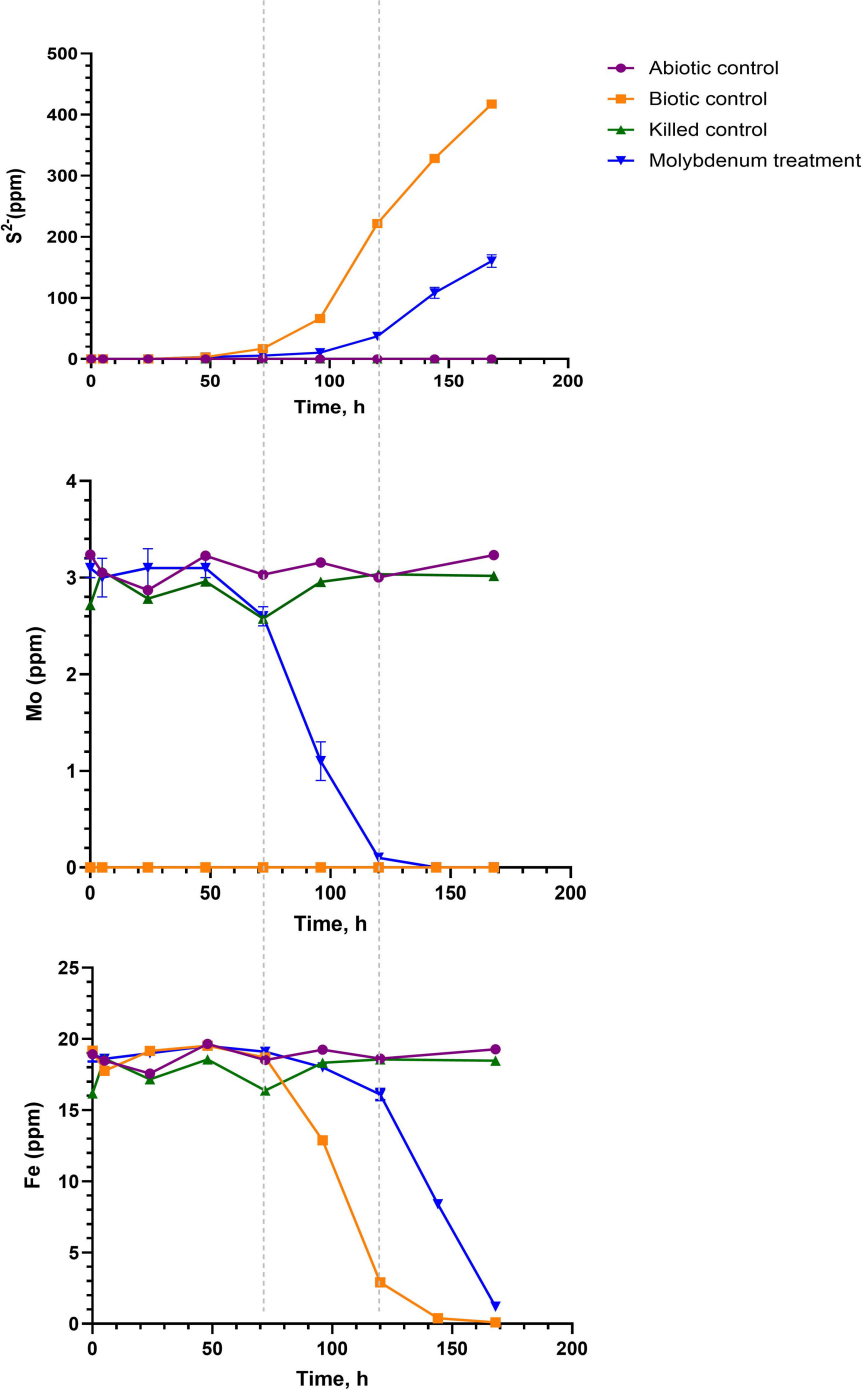
Sulfide, iron, and molybdenum dynamics in an experiment with a microbial community enriched from MW 3. The vertical dashed lines indicate the two stages of the experiment. Error bars for Mo treatment represent triplicate measurements, and in many cases, the error is smaller than the point.

The addition of Mo to the enriched culture suppressed sulfide production in the Mo-treatment group compared to the biotic control, while the killed control and abiotic controls did not show sulfide production (Fig. 4). Similar results have been observed for other SRB, where Mo addition led to the inhibition of sulfate reduction (7, 19). The lag phase of the microbial growth was ~50 h, regardless of the presence of the Mo (Fig. 4). In this study, biotic control showed only one stage of development with a black precipitate formation at 96 h, which was also associated with a decrease in Fe concentration (Fig. 4; Fig. S6). In the Mo-treatment experiments, we observed two phases within the microcosms. The first phase was characterized by the development of a red-brown color at 72 h, which was accompanied by a decrease in Mo concentration (Fig. 4). At approximately 100 h, the concentration of Fe decreased in the Mo-treatment experiments, accompanied by the formation of a black precipitate. The red-brown color was observed in previous studies (19, 21) and can be attributed to the reduction of molybdate, the formation of a molybdate-sulfate complex in the periplasm of the cell, and the accumulation of molybdenum sulfide extracellularly or at the surface of the cells (19). Biotic and abiotic mechanisms of Mo incorporation into Fe-monosulfide or pyrite have been proposed (23, 55, 56). The abiotic Fe-sulfide pathway depends on the oxidation state of Mo and requires reduction from Mo(VI) to Mo(IV) (57). SRB reduces molybdenum to Mo(V) and forms the periplasmic Mo(V)-sulfur complex (19). In our study, the SEM analysis uncovered an absence of Mo in the precipitates, which were predominantly composed of C, O, Fe, and S (Fig. S7). Overall, this suggests that the Mo is associated with the bacterial cells, not extracellular precipitates or aqueous Mo-S species.

### Microbial response to molybdenum

**Fig 5:**
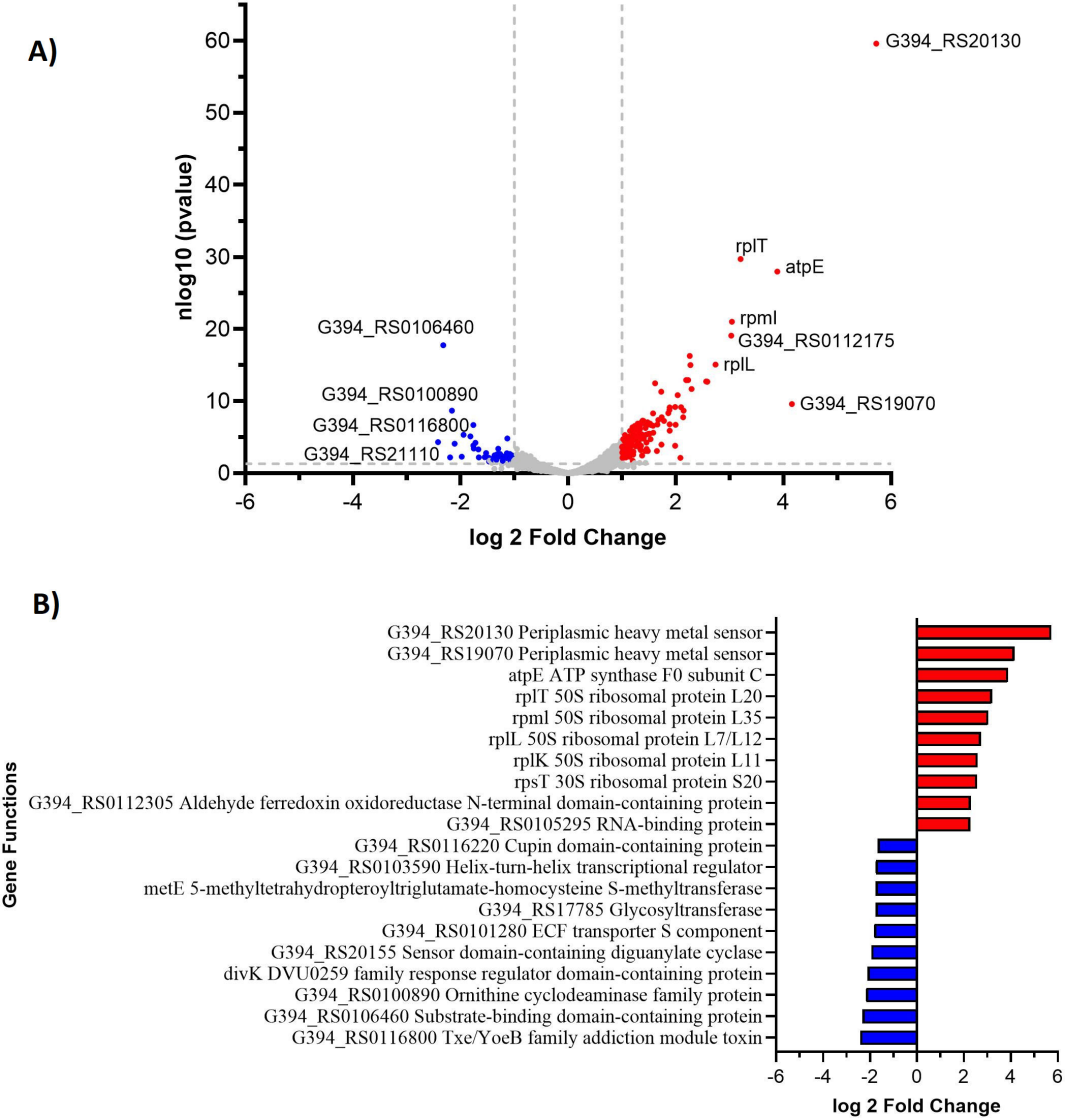
Volcano plot (**A**) and log2FC (**B**) of microcosm with and without the addition of Mo. Positive and negative values show upregulation and downregulation of the genes, respectively.

To determine which organisms were most active during Mo treatment, we performed RNA-seq of the incubations during molybdate reduction. We identified two organisms that had the highest transcript abundance: *Desulfomicrobium escambiense* and *Clostridium sporogenes*. In the absence of Mo treatment, reads mapping to the *C. sporognes* genome accounted for 33%–44% of the total reads, and *D. escambiense* accounted for 20%–41%. With the Mo amendment, the *C. sporongnes* transcript abundance decreased to 5%, whereas *D. escambiense* reads increased to 46%. These results suggest that *D. escambiense* may have a role in the decrease of Mo concentration observed in the microcosms. In the Mo-treatment experiment, 143 genes (log2FC > 1; *P* < 0.05) were upregulated in *D. escambiense* in response to Mo (Fig. 5). The most highly differentially expressed upregulated genes included two periplasmic heavy metal sensors with log2FC > 4. The two most highly upregulated genes were G394_RS20130 and G394_RS19070, both predicted to encode periplasmic heavy metal sensors based on Hidden Markov Model domain inference. G394_RS20130 shares sequence similarity with the Spy/CpxP family of stress response proteins (58), whereas G394_RS19070 is homologous to the zinc resistance-associated protein ZraP (59). These proteins are known to detect and respond to extracytoplasmic stress in enteric bacteria through chaperone-like activity and modulation of the CpxRA and ZraSR two-component regulatory systems. Other upregulated genes were *atpE*, ATP synthase, and a number of ribosomal proteins (Fig. 5).

**Fig 6:**
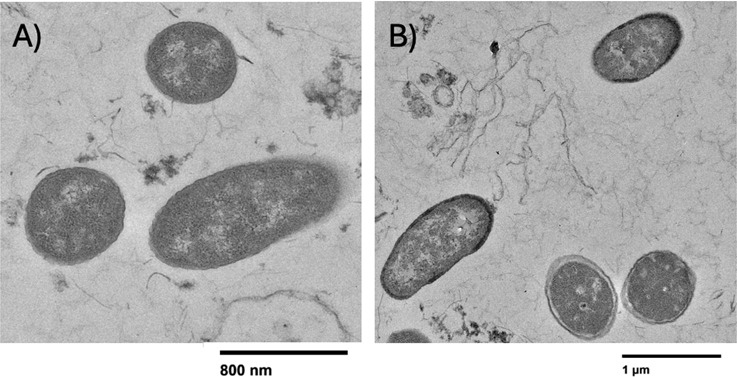
TEM image of the enriched culture from the MW-3 sample before (**A**) and after (**B**) the molybdenum treatment. An opaque material was observed in the bacteria in Mo-treatment experiments, likely due to Mo-S accumulation in the cell periplasm.

Mo uptake has been shown for several gram-negative bacteria (60) and involves the ModABC transporter, molybdopterin cofactor (Moco) or Fe-Mo cofactor (FeMoco) (61). Interestingly, the differential expression of Mo-uptake genes *moaC, modA, moaA,* and *modB* (60) was not significant, indicating that Mo uptake may not be a predominant mechanism influencing the Mo concentrations observed in the experiments. Instead, detoxification may be the mechanism by which Mo is transformed by *D. escambiense*. The detoxification mechanism is activated at high metal concentrations and is a two-step process. First, a metal sensor protein activates and shuts off metal uptake, followed by up-regulation of efflux or detoxifying system (62). The metal efflux occurs through cation diffusion facilitator (CDF) or P-type ATPase mechanisms and was characterized for other metals (19, 63, 64). In this study, we observed the upregulation of several periplasmic heavy metal sensors, which may be responsible for sensing the high concentration of extracellular Mo and activation of the *atpE* ATP synthase that may play a function of exporter similar to P-type ATPase mechanisms. Our data are consistent with a similar model observed for Co^2+^/Mg^2+^ (65). The activation of the Mo-detoxifying over uptake mechanism was evidenced by TEM analysis, which showed nanocrystalline structures (presumably MoS_2_) in the cell periplasm after Mo treatment (Fig. 6). The mineralogy or composition of nanocrystals is not known in these experiments, but the results are visually consistent with previous experiments with *Desulfovibrio desulfuricans* DSM 642 and *Desulfovibrio gigas* ATCC 19364 that did confirm poorly crystalline Mo-S species through energy dispersive X-ray analysi*s* (19).

### Bioremediation implications

Sulfate-reducing bacteria have proven useful for bioremediation at a wide range of sites that release metals and metalloids to groundwater (25, 53). Particularly for low pH environments like acid mine drainage and wastewater, sulfate reduction is a key mechanism to sequester metals and metalloids like As, Zn, Cu, Ni, Hg, and Cr (53). In environments where pH > 7, a combination of sulfate-reducing and sulfur/sulfide-oxidizing bacteria is present (53). It is expected that internal and external sequestration mechanisms for metals persist, and hence, the same functional bioremediation potential exists even at higher pH. With respect to Mo, bioremediation through precipitation of Mo sulfides (analogous to Fe sulfide formation) either by direct or indirect means poses a challenge since Mo can suppress sulfate reduction (21). In this study, SRB were depleted in high-Mo wells, but not absent. In particular, the presence and function of *D. escambiense* for SRB-based Mo sequestration could still be a viable remediation pathway. The activation of the putative Mo-detoxification mechanism by *D. escambiense* makes this strain tolerant to Mo in groundwater and opens a potential for the development of the geochemistry-informed bioremediation technologies of Mo-contaminated groundwater.

There are several considerations necessary for scaling from microcosm to field applications. First, biotransformation often occurs in heterogeneous environments, with steep energy gradients, niche spaces, and complex microbial interactions. The approach here of identifying a singularly active species has the advantage of identifying fundamental biochemical pathways of transformation. However, it does not address complex microbial interactions that are needed to inform sustainable development of communities *in situ* or engineering of synthetic communities (66). For example, bacteria are more frequently studied as the dominant players in biotransformation processes of metals and metalloids, hydrocarbons, and chlorinated compounds, but other organisms that are under selected or unidentified using 16S rRNA survey techniques can perform roles for primary and syntrophic biotransformation of contaminants (67, 68). Niche players and the distributed functions across the native community (e.g., cross-feeding, syntrophic relationships) are key relationships needed to scale microcosms to field-scale applications.

Second, further studies are needed to elucidate community relationships and ways that enrichment methods could select for the most effective consortia for Mo sequestration. In this study, *Desulfobacterales, Desulfovibrionales, and Desulfuromonadales* were observed at relative abundances > 1% in groundwater, but *Desulfomicrobium* was the most abundant SRB observed during enrichment. It is well established that carbon substrates (e.g., alcohols, organic acids, and sugars) could produce different microbial assemblages during enrichment (69). This would be important to understand when scaling these results and biostimulating SRB communities *in situ*.

Third, the potential for bioremediation is also predicated on the extent to which these results are generalizable to other sites. SRB are ubiquitous in subsurface geological environments under anoxic conditions. However, further research is needed to establish the extent to which periplasmic sequestration mechanisms are prevalent in the wider SRB taxa that include seven phylogenetic lineages (70). *Desulfovibrio* has shown tolerance to molybdate up to 2 mM (~190 ppm Mo) with *D. gigas* and *D. vulgaris* more sensitive to Mo as a respiration inhibitor compared with *D. desulfuricans* (19). In this study, *Desulfomicrobium escambiense* showed a similar response to that of *Desulfovibrio* (~30% suppression of sulfate reduction), suggesting that the mechanism could be more common outside of *Desulfovibrio*.

Finally, it is important to recognize the ways in which these results influence field-scale fate, transport, and transformation of Mo. A nuance here is that Mo is sequestered in the cell as opposed to extracellular mineral precipitates like those in other metal sequestration strategies or complete mineralization like those in (chlorinated) hydrocarbon efforts. Therefore, long-term sequestration is influenced by factors that generally influence bacterial cell transport (biofilm development, interaction with mineral surfaces, flow rates) as well as the stability and potential phase transformation (e.g., crystallization) of the Mo-precipitates in the cell after cell death. Sequestration of Mo through sulfate reduction has been verified in column reactors containing *Desulfovibrio desulfuricans,* where greater than 99% removal efficiencies were observed at sublethal Mo concentrations (21), providing some evidence of immobilization. However, there are many outstanding questions about geochemical perturbations in redox conditions or pH regimes that occur in different hydrogeological environments that make this an area of active research.

## Data Availability

This Targeted Locus Study project has been deposited in GenBank and the Sequence Read Archive (SRA) under BioProject PRJNA1339003.

## SUPPLEMENTAL MATERIAL

Supplemental figures aem.00988-25-s0001.pdf Figures S1 to S7.

ASM does not own the copyrights to Supplemental Material that may be linked to, or accessed through, an article. The authors have granted ASM a non-exclusive, world-wide license to publish the Supplemental Material files. Please contact the corresponding author directly for reuse.
