# Supplementary material for: Microbial community diversity and geochemistry inform bioremediation of molybdenum-contaminated groundwater
Source: Appl Environ Microbiol. 2025 Dec 5;91(12):e00988-25. doi: 10.1128/aem.00988-25 (PMC12724210; doi:10.1128/aem.00988-25)
Supplement: Supplemental figures — Figures S1 to S7. [file aem.00988-25-s0001.pdf]

Microbial Community Diversity and Geochemistry Inform Bioremediation of Molybdenum Contaminated Groundwater

Natalia Malina[1,2] *Rodney Tollerson II*[3], *Shifat J. Monami*[1], *Elyssa Rivera*[1], *Ming-Kuo Lee*[1], *Laura D. Bilenker*[1] *Ann Sullivan Ojeda*[1]*,

[1] Auburn University, Department of Geosciences, 2050 Beard-Eaves Memorial Coliseum, Auburn, Alabama, 36849 USA

[2] Florida Atlantic University, Department of Chemistry and Biochemistry, Boca Raton, Florida, 33431, USA

[3] Auburn University, Department of Biological Sciences, Auburn, Alabama 36849, USA

*corresponding author

Table of Contents

1. Study Site Overview 2
a. Site Map 2
b. Hydrochemical Facies 3
2. Diffusive Microbial Sampler Construction 4
3. Microbiological diversity 5
4. Molybdenum sequestration experiment 6
5. SEM analysis 7
6. REFERENCES 8

1. Study Site Overview

a. Site Map

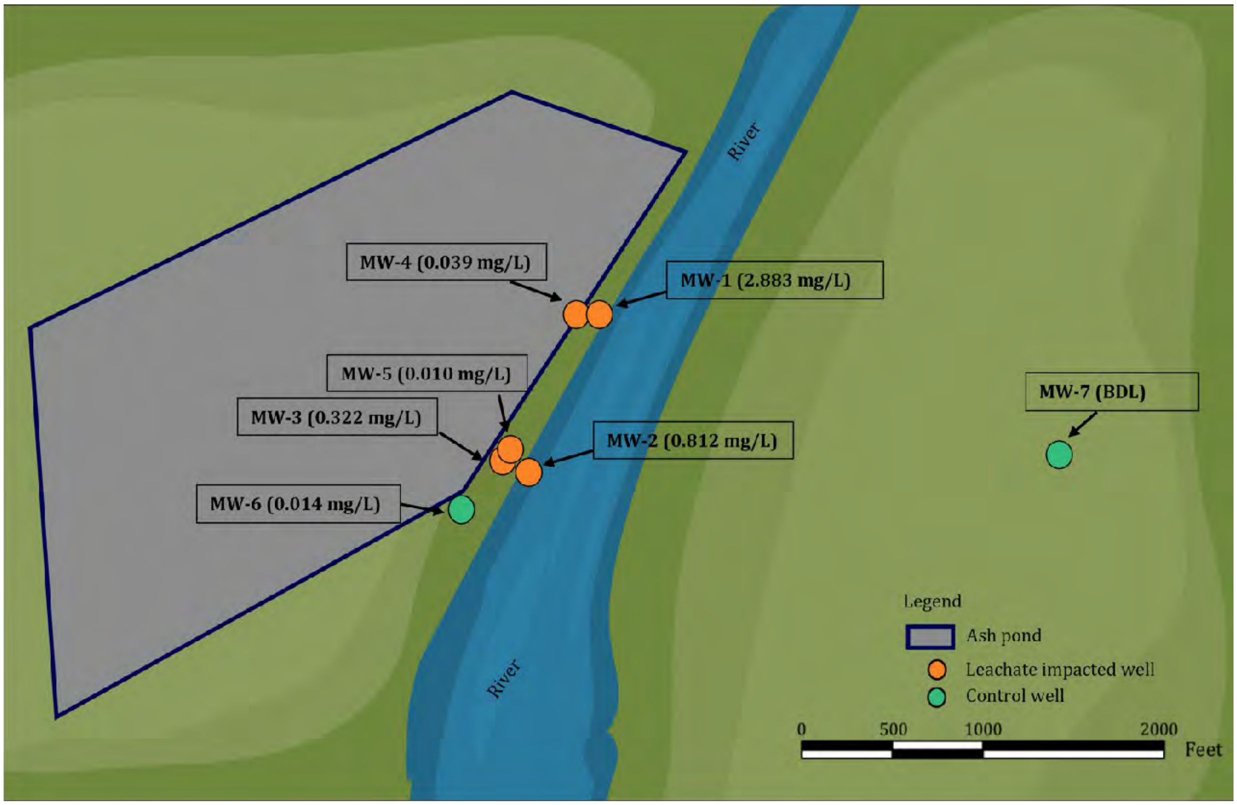

Figure S1. Schematic of the study site depicting ash pond and monitoring wells within the leachate plume (orange) and control wells (green). Wells are annotated with the Mo concentration in mg/L. BDL = below detection limit. Groundwater flow is left to right toward the river; river flow is from the top of the image to the bottom.

b. Hydrochemical Facies

A Piper diagram (Piper, 1944) was constructed using Geochemist's Workbench (Bethke, 2022) to investigate the hydrochemical facies of the groundwater at the site (Figure S2).

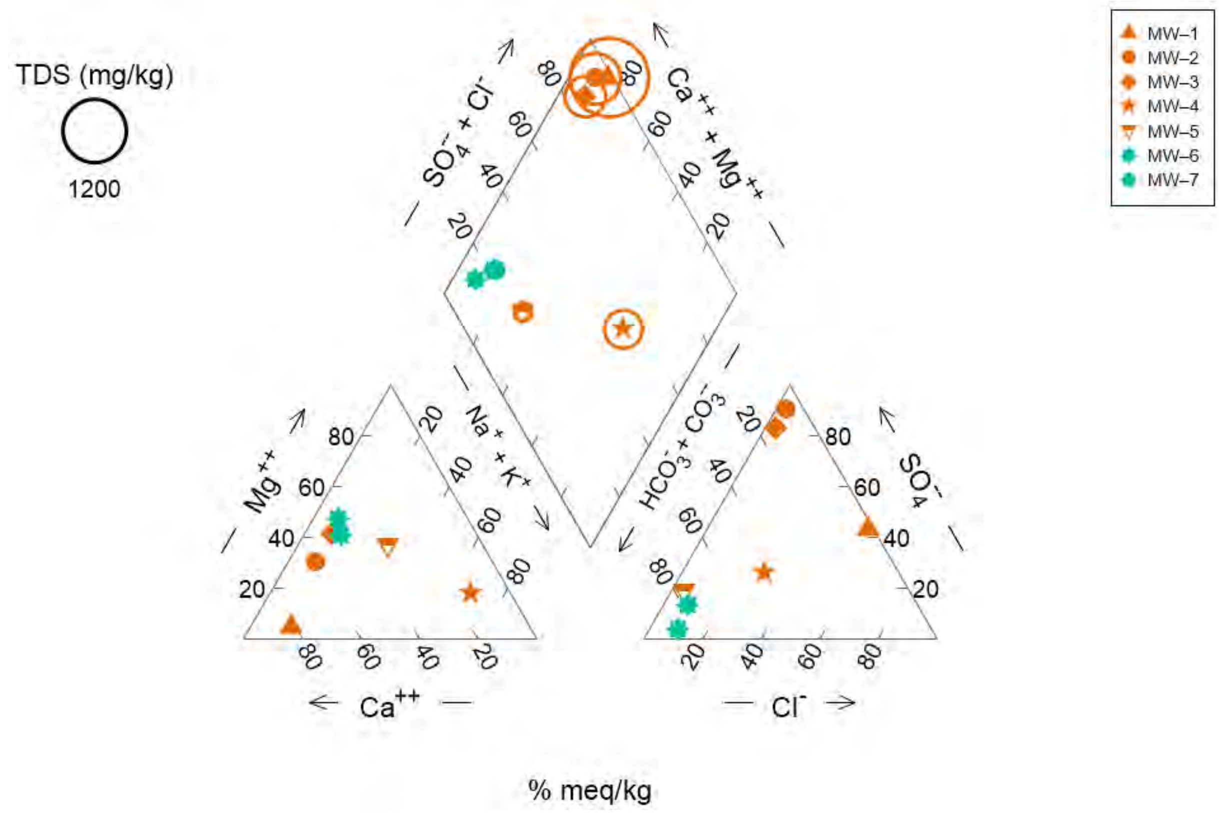

Figure S2. Piper diagram showing hydrochemical facies of the groundwater at the study site. Green symbols indicate control wells, while orange circles are the leachate-impacted wells. The circle around each symbol in the center diagram is proportional to the amount of total dissolved solids (TDS).

2. Diffusive Microbial Sampler Construction

The diffusive microbial sampler (DMS) was constructed using two Snap Samplers (QED Environmental, Dexter, MI) connected in sequence. Key features of the Snap Sampler included 40mL glass volatile organic analysis (VOA) vials and 302 stainless steel bottle spring with perfluoroalkoxy (PFA) coating, pictured on the left panel in Figure S3. Nylon mesh (Component Supply, 85µm mesh opening) was used to craft a package by heat treating the seams, and to it was added ~8g of sterilized sand (Ward Scientific) (Figure S3). The package was suspended in the vial by threading the top of the package through the spring contained inside of the vial. During deployment, the package was suspended inside of the vial as a substrate for microbes to culture.

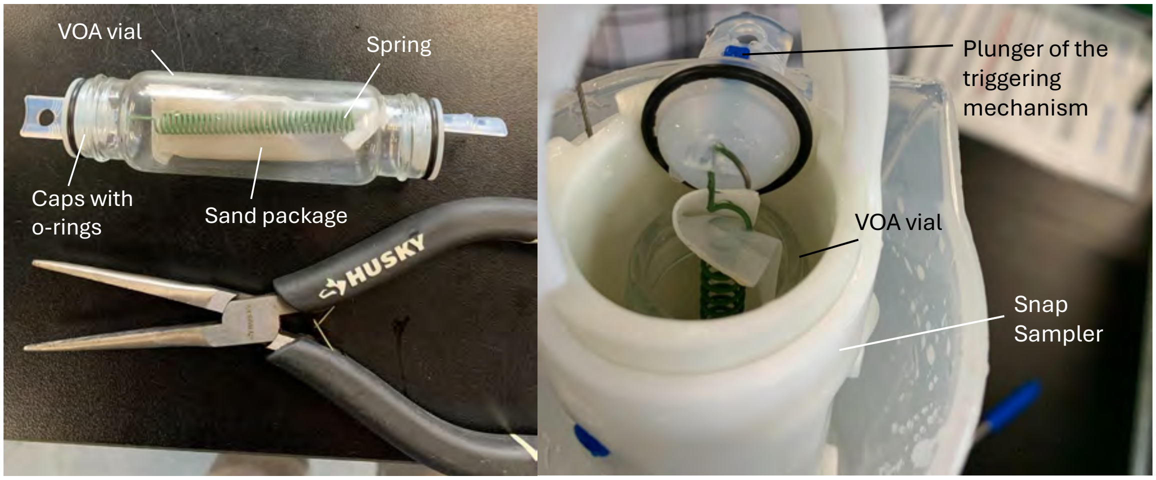

Figure S3. Image of the modified VOA vial used in the DMS (left), and image of the vial and package in the “open” position as it would be down-hole.

3. Microbiological diversity

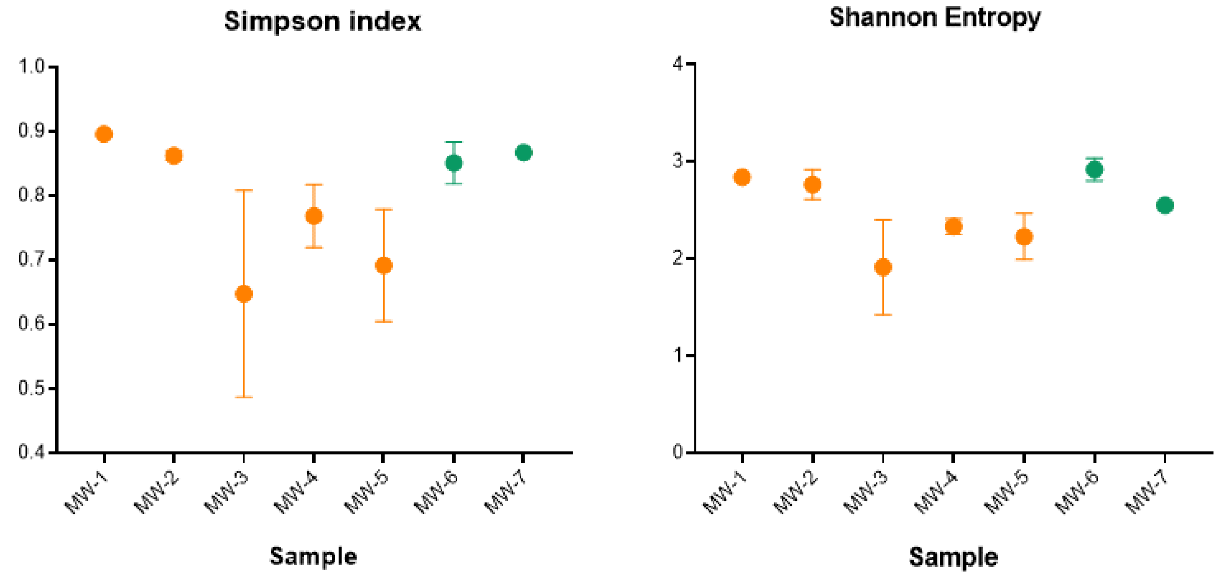

Figure S4. Alpha-diversity metrics, Simpson index (left) and Shannon entropy function (right), calculated at the order taxonomic level. Samples are color coded as wells within the plume (orange) and control samples (green). Error bars represent the range between duplicate samples. In some cases, error bars are smaller than the point.

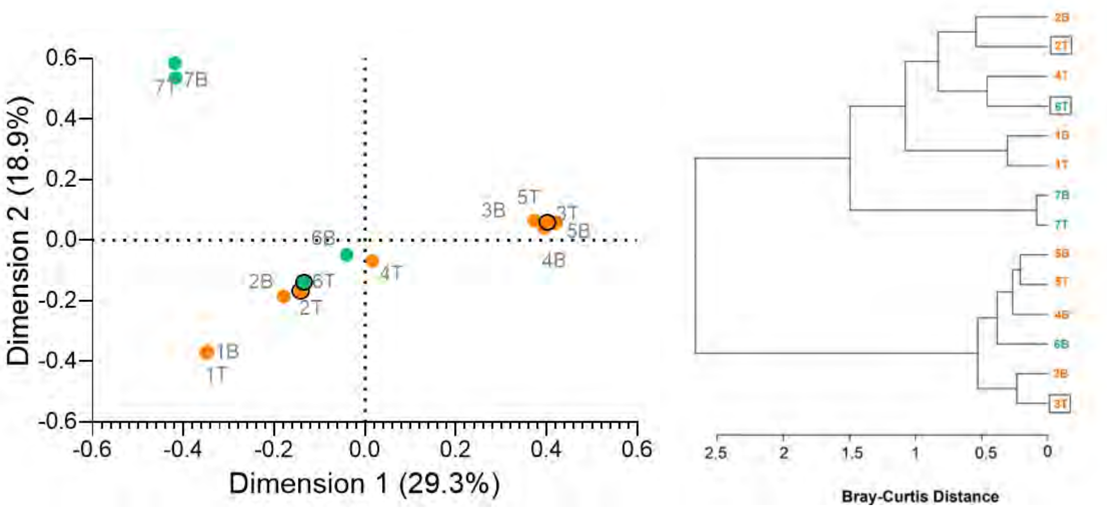

Figure S5. Ward clustering method shows beta-diversity (right) and community clusters based on the Bray-Curtis distance between samples (right). Samples were color coded as wells within the plume (orange) and control wells (green). Samples with a black outline did not close successfully downhole using the DMS.

4. Molybdenum sequestration experiment

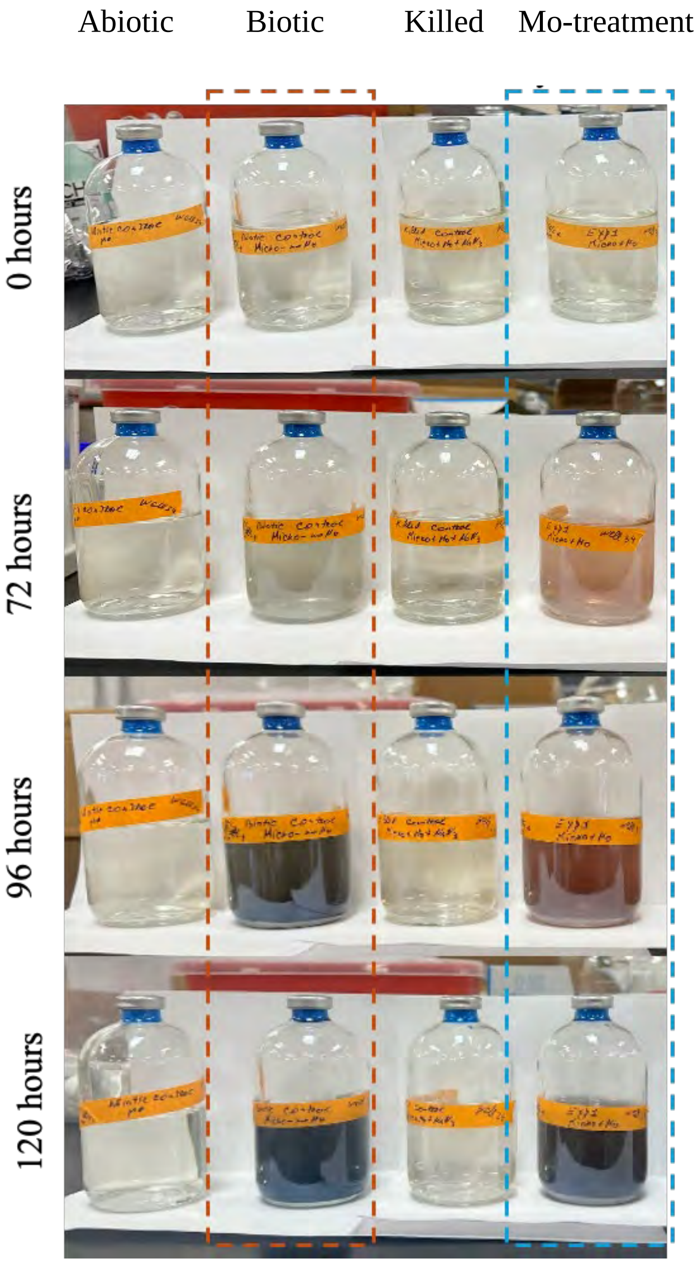

Figure S6. Images of microcosm experimental bottles for abiotic, biotic (red box), and killed controls as well as Mo-treatments (blue box)

5. SEM analysis

Sample preparation was conducted in an anaerobic chamber. The serum bottle solution was transferred to the 50 ml centrifuge tube, and the supernatant was carefully removed using pipettes. Deionized water was added to the centrifuge tube, manually shaken, and centrifuged for 15 min at 4000 rpm. The precipitate washing was repeated thrice. Then, the precipitates were air-dried for several days in the anaerobic chamber until it was completely dried. The dried samples were mounted onto an SEM pin stub using carbon tape to provide electrical conductivity and stable positioning. To enhance imaging quality and surface conductivity, the samples were gold-coated using a sputter coater. The prepared samples were then analyzed through backscattered electron imaging to study compositional variations and SEM-EDS point analysis to examine the elemental composition of the precipitates with an accelerating voltage of 15 kV and a beam current set to 30 µA.

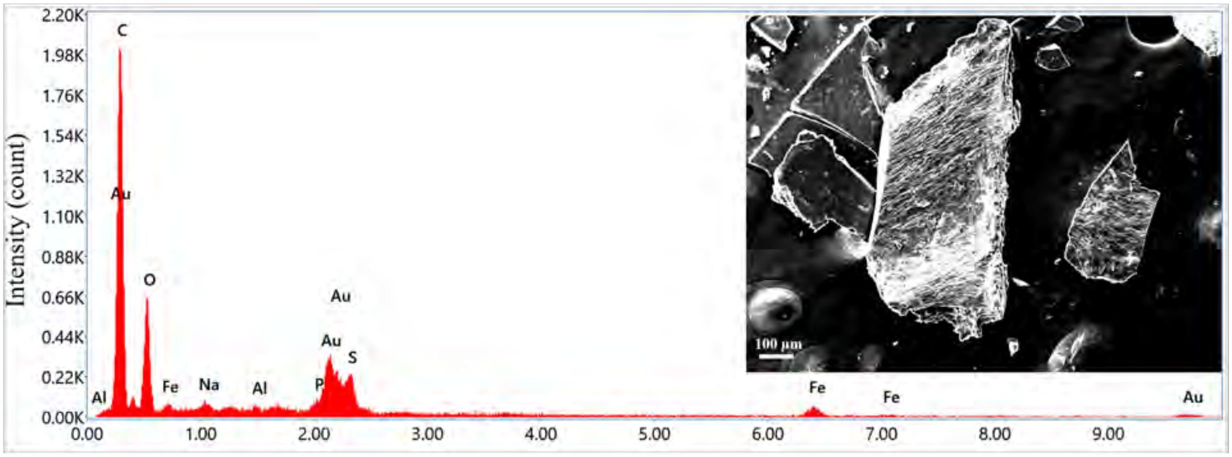

Figure S7- BSE image and EDS spectrum of the precipitates in the Mo-treated experiments at 120 hr.

6. REFERENCES

Bethke, C.M., 2022. Geochemical and biogeochemical reaction modeling. Cambridge university press.

Piper, A.M., 1944. A Graphical Procedure in the Geochemical Interpretation of Water-Analyses. Trans. Am. Geophys. Union 914–928.
